# Suppression of CPSF6 Enhances Apoptosis Through Alternative Polyadenylation-Mediated Shortening of the *VHL* 3′UTR in Gastric Cancer Cells

**DOI:** 10.3389/fgene.2021.707644

**Published:** 2021-09-14

**Authors:** Xinglong Shi, Keshuo Ding, Qiang Zhao, Pengxiao Li, Yani Kang, Sheng Tan, Jielin Sun

**Affiliations:** ^1^Ministry of Education Key Laboratory for Systems Biomedicine, Shanghai Center for Systems Biomedicine, Shanghai Jiao Tong University, Shanghai, China; ^2^Department of Pathology, School of Basic Medicine, Anhui Medical University, Hefei, China; ^3^Department of Pathology, The First Affiliated Hospital of Anhui Medical University, Hefei, China; ^4^School of Biomedical Engineering, Shanghai Jiao Tong University, Shanghai, China

**Keywords:** alternative polyadenylation, gastric cancer, apoptosis, CPSF6, VHL

## Abstract

Alternative polyadenylation (APA) is an important RNA post-transcriptional process, which can generate diverse mRNA isoforms. Increasing evidence shows that APA is involved in cell self-renewal, development, immunity, and cancer. CPSF6 is one of the core proteins of CFIm complex and can modulate the APA process. Although it has been reported to play oncogenic roles in cancer, the underlying mechanisms remain unclear. The aim of the present study was to characterize CPSF6 in human gastric cancer (GC). We observed that CPSF6 was upregulated in GC. Knockdown of CPSF6 inhibited proliferation and enhanced apoptosis of GC cells both *in vitro* and *in vivo*. Global APA site profiling analysis revealed that knockdown of CPSF6 induced widespread 3′UTR shortening of genes in GC cells, including *VHL*. We also found CPSF6 negatively regulated the expression of VHL through APA and *VHL* short-3′UTR isoform enhanced apoptosis and inhibited cell growth in GC cells. Our data suggested that CPSF6-induced cell proliferation and inhibition of apoptosis were mediated by the preferential usage of poly(A) in *VHL*. Our data provide insights into the function of CPSF6 and may imply potential therapeutic targets against GC.

## Introduction

Gastric cancer (GC), one of the top five prevalent cancers, is the third leading cause of cancer mortality across the world, owing to its poor prognosis and diagnoses only at advanced stage with median overall survival less than 1 year (Zhang and Zhang, 2017; Smyth et al., 2020). Many predisposing factors, such as *Helicobacter pylori* infection, age, environmental factors, dietary habits, and so on, assist in the development of GC (Chia and Tan, 2016; Petrovchich and Ford, 2016). Apart from that, genetic alterations are also reported to be involved in GC progression. Abnormal expression of *HER2*, *CDH1*, *TP53*, *FGFR*, *MET*, and other genes are frequently found in different GC types (Oue et al., 2019). The regulation of GC development and progression seems particularly complex; hence, a deeper understanding of the pathogenesis mechanism of GC at the molecular level may be beneficial to identify potential therapeutic targets.

Alternative polyadenylation (APA) is an RNA post-transcriptional process that produces distinct messenger RNA (mRNA) isoforms of a single gene through dictating the length of 3′ untranslated regions (UTRs). Modification of 3′UTR influences mRNA stability, translation, and cellular localization, as 3′UTRs provide major binding sites for protein factors or microRNAs (miRNAs) (Andreassi and Riccio, 2009; Fabian et al., 2010). Recent researches demonstrate that APA can affect various biological functions, including cell self-renewal, differentiation, activation of human immune cells, neuronal activation, and so on (Flavell et al., 2008; Sandberg et al., 2008; Ji and Tian, 2009; Lackford et al., 2014). Of note, APA is also involved in cancer. For example, *Pik3ap1* displayed preferential loss of signal in the proximal 3′UTR in APC and APN cancer subtypes, uniform degradation in LPC cancer subtype, and no change in mature B cells (Singh et al., 2009). Another study also reported that lung (LUSC and LUAD), uterine (UCEC), breast (BRCA), and bladder (BLCA) cancers possessed the highest amount dynamic APA events than the other tumor types, such as kidney renal clear cell carcinoma (KIRC) and head and neck squamous cell carcinoma (Xia et al., 2014).

APA can be determined by over 80 proteins, and the core complex contains four subcomplexes: CPSF, CSTF, CFI, and CFII (Tian and Manley, 2017). Some of them are reported to play an important role in tumorigenesis. It was reported that CSTF2 enhanced oncogenic and metastatic abilities in urothelial carcinoma of the bladder cells (Chen et al., 2018). Intriguingly, NUDT21 (CFIm25) showed a dual role in cancers. For example, NUDT21 was downregulated and functioned as tumor suppressor in glioblastoma, hepatocellular carcinoma, and ovarian cancer (Masamha et al., 2014, 2016; Tan et al., 2018). However, other groups uncovered that knockdown of NUDT21 significantly inhibited cell proliferation and tumorigenicity (Lou et al., 2017; Zhang and Zhang, 2018). These observations implicate sophisticated regulation of APA factors. Meanwhile, how APA takes part in tumor formation is still unclear.

The polyadenylation complex cleavage factor I (CFIm) preferentially binds to UGUA subsequences in pre-mRNAs and controls 3′UTR length, which consists of NUDT21, CPSF6, and CPSF7 (Gruber et al., 2012). In addition, the recombinant NUDT21-CPSF6 subunit complex could also show cleavage activity *in vitro*, which was similar with full CFIm complex (Rüegsegger et al., 1998). As described earlier, NUDT21 regulated tumor growth negatively or positively (Tan et al., 2018). Also, to date, as its partner, the description of CPSF6’s function was mainly in HIV infection (Lee et al., 2010; Bejarano et al., 2019). Recent reports also discovered the oncological function of CPSF6, but not in an APA regulatory perspective (Binothman et al., 2017; Zhang et al., 2020). As one of the APA factors, CPSF6 plays an important role in regulation of 3′UTR length. It is reported that knockdown of CPSF6 significantly upregulated the usage of proximal PAS in C2C12 myoblast cells (Li et al., 2015). Besides that, another group also found that knockdown of CPSF6 led to a systematic and transcriptome-wide shift toward proximal poly(A) sites in HEK293 cells (Gruber et al., 2012; Martin et al., 2012). So, it is necessary to discover the roles of CPSF6 in GC progress.

Herein, to expand our knowledge on CPSF6 biological roles, we investigated the phenotypes of CPSF6 knockdown GC cells. We found CPSF6 promoted tumor growth and inhibited apoptosis in GC. Furthermore, we demonstrated CPSF6 negatively regulated von Hippel Lindau (*VHL*), the gene encoding a tumor suppressor, through selective poly(A) usage, thus contributing to tumor growth in GC. Our data provide insights into the function of CPSF6 and may imply potential therapeutic targets.

## Materials and Methods

### Cell Lines and Cell Cultures

Human normal gastric cell line GES1 and GC cancer cell lines (AGS, BGC-823, MKN-28) were purchased from the Chinese Academy of Sciences cell bank (Shanghai, China). All cells were cultured in RPMI-1640 medium (Gibco, United States) supplemented with 10% fetal bovine serum (FBS, Gibco, United States) and penicillin–streptomycin solution (Sangon, China) at 37°C in a 5% CO_2_ humidified atmosphere.

### Quantitative Real Time RT-PCR (qRT-PCR)

Total RNA was isolated using Trizol (Life Technologies, United States) and reverse transcribed with PrimeScript RT Reagent Kit with gDNA Eraser (Perfect Real Time) (Takara, Japan). Real-time qRT-PCR analysis was performed using TB Green Premix Ex Taq II (Tli RNaseH Plus) (Takara), according to the manufacturer’s protocol. The primers used for RT-qPCR analysis are listed as follows: *VHL* 5′-CCCGTATGGCT CAACTTCG-3′ and 5′-GGTTAACCAGAAGCCCATCG-3′; *GAPDH* 5′-CACAGTCCATGCCATCACTG-3′ and 5′-CTTGGCAGCGCCAGTAAG-3′. The expression of genes was normalized to *GAPDH.*

### Western Blot Analysis

Cells were lysed in ice-cold RIPA Lysis and Extraction Buffer (Thermo, United States) supplemented with protease (Roche, Switzerland) and phosphatase inhibitor cocktail (Roche). The protein concentrations were determined using BCA Protein Assay Kit (Pierce, United States). Protein lysate was separated by 10% or 12% sodium dodecyl sulfate polyacrylamide gel electrophoresis system (SDS-PAGE) and transferred onto PVDF membranes (Millipore, United States). The membrane was incubated in blocking buffer (1 × TBST with 5% BSA) for 2 h at room temperature and then incubated in diluted primary antibodies: anti-CPSF6 (1:1,000; Abcam, United Kingdom), anti-VHL (1:1,000, Sangon), anti-GAPDH (1:1,000; Proteintech, China), anti-β-actin (1:5,000; Sigma, United States), and anti-β-tubulin (1:1,000; Yeasen, China), and secondary antibody: peroxidase-conjugated goat anti-rabbit IgG (H+L) (1:5,000; Yeasen). The membrane was visualized using ECL start Western Blotting Substrate (GE Healthcare Life Sciences, United States). The intensities of the bands were qualified by ImageJ (National Institutes of Health, United States).

### Generation of Stable Cell Lines

The lentiviral vector pLKO.1-puro-shRNA-*CPSF6* was constructed for CPSF6 knockdown as described previously (Tan et al., 2021). The lentiviral vectors pCDH-PGK-*VHL*-CDS-short 3′UTR-SV40-NeoR and pCDH-PGK-*VHL*-CDS-long 3′UTR-SV40-NeoR were constructed for VHL overexpression. 293T cells were transfected for the lentiviral production. The supernatant was harvested after 48 and 72 h, respectively. Then AGS and BGC-823 cells were infected by supernatant for 72 h. Finally, cells were maintained in the presence of puromycin (2 μg/ml) and G418 (600 μg/ml or 1.2 mg/ml) for 2 weeks.

### Cell Proliferation and Cell Viability Assays

For cell proliferation assay, cells (1 × 10^5^ cells per well) were plated into 6-well plates. After a certain period of time, count the cell number of fields. Then, calculate the cell number of each well. For cell viability assay, cells (1 × 10^3^ cells per well) were plated into 96-well plates. Five days later, 20 μl of MTT solution (5 mg/ml) was added into each well and cells were cultured for 2 h, and cells were rinsed by PBS and 100 μl DMSO was added into each well. Then the absorbance at 570 nm wavelength was examined.

### Colony Formation Assays

GC cells were seeded into 6-well plates (500–1,000 cells/well) and cultured for 2 weeks. Cells were fixed with 4% methanol, stained with crystal violet (0.1%), and photographed.

### Cell Apoptosis Assays

The apoptosis rate was evaluated using the Annexin V-FITC/PI Apoptosis Detection kit (Vazyme, China) according to the instructions from the manufacturer. The cells were seeded into 6-well tissue culture plates (1–4 × 10^5^ cells/well). After cultured in serum-free medium for 36 h, the cells were collected, washed with ice-cold PBS, and resuspended in 500 μl binding buffer. Then, 5 μl Annexin V-FITC and 5 μl PI were added to the buffer and incubated at room temperature for 10 min in the dark. Cells were analyzed by BD LSRFortessa (BD Biosciences, United States) within 1 h. The TUNEL staining assay was performed using Fluorescein (FITC) Tunel Cell Apoptosis Detection Kit (Servicebio, China) according to the manufacturer’s instructions.

### Xenograft Model *in vivo*

The design and protocol of *in vivo* experiments were approved by the Institutional Animal Care and Use Committee, Shanghai Jiao Tong University. For tumor growth assay, GC cells were resuspended in PBS/Matrigel Matrix mix at 1:1 ratio. Then 3 × 10^6^ cells in 125 μl solution were injected into the flank of 4-week-old athymic male nude mice. Tumor volumes were calculated as follows: volume = ½ (*L* × *W*^2^), where *L* is the length and *W* is the width. Tumor weight was measured when the mice were executed. The IHC staining of xenograft tumors was performed following the manufacturer’s instruction. IHC labeling analysis was reviewed and scored by two independent pathologists.

### Dual Luciferase Reporter Assays

The short or the long 3′UTR of the *VHL* gene was cloned into reporter plasmid psiCHECK2 (Promega, United States). GC cells were transfected with the reporter vector in 24-well plates (200 ng/well) using Lipofectamine 3000 (Invitrogen, United States). After 48 h, cells were collected and assayed using dual luciferase assay according to the manufacturer’s protocol (Transgen, China).

### 3T-Seq Analysis

3T-seq analysis was performed as described previously (Lai et al., 2015; Tan et al., 2018). Briefly, 50 μg total RNA was incubated with M280 beads, which were bound by Bio-oligo dT (Lou et al., 2017). After cDNA synthesis, the 3′UTR fragments were released by *Gsu I* digestion and then subjected to deep sequencing. The filtered reads were mapped to UCSC human reference genome (hg19) with bowtie2 (Langmead and Salzberg, 2012). The 3′UTR alteration for each gene in the cell lines was detected by linear trend test (the FDR-adjusted *p*-value < 0.05) (Fu et al., 2011). These sequence data have been submitted to the EMBL-EBI databases under accession number E-MTAB-9980 and available in the ArrayExpress database.^1^

### Statistical Analysis

All experiments were repeated at least three times. The results of this study were analyzed with GraphPad statistical software (GraphPad Software, United States) and shown as mean ± SD. We downloaded the expression data of CPSF6 in normal and GC tissues from The Cancer Genome Atlas (TCGA) database^2^ and selected frozen sample data for analysis. To investigate the differential expression of CPSF6 between normal and GC tissues of distinct cancer stages or nodal metastasis status in TCGA, we used the website tool^3^.

## Results

### CPSF6 Is Upregulated in GC

**FIGURE 1:**
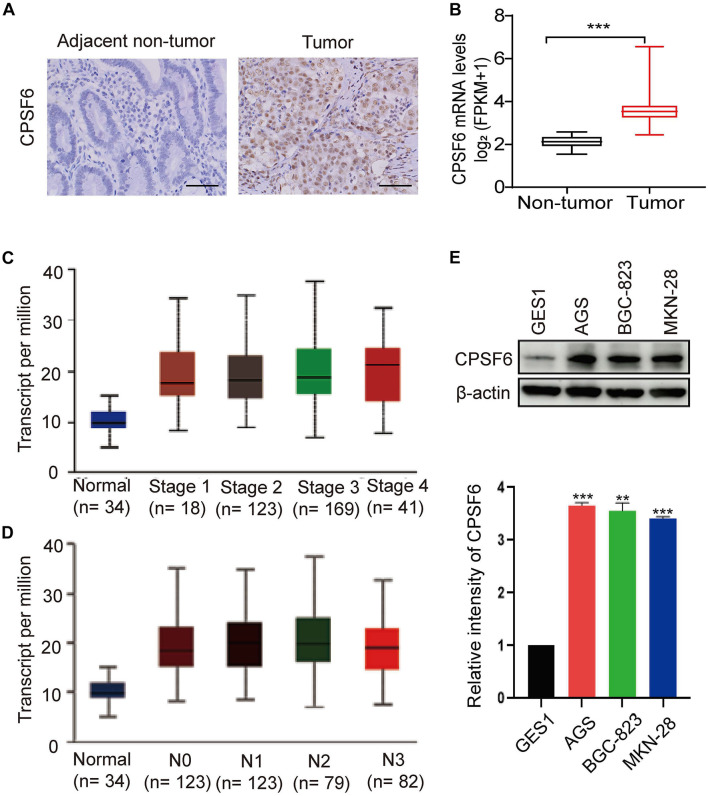
Expression of CPSF6 was upregulated in GC. **(A)** The IHC staining assay of CPSF6 in GC and adjacent tissues of the tumor (scale bar: 100 μm). **(B)** Analysis of CPSF6 mRNA levels of GC (*n* = 352) and non-tumor tissues (*n* = 32) in TCGA. **(C)** Expression of CPSF6 in GC based on individual cancer stages in TCGA. **(D)** Expression of CPSF6 in GC based on nodal metastasis status in TCGA. **(E)** Western blot assay for detecting CPSF6 expression in different cell lines. **0.001 < *p* < 0.01; ***p < 0.001.

In this study, we first analyzed the CPSF6 levels using IHC assay in GC tissues and the paired non-tumor tissues. IHC staining assay showed that CPSF6 was upregulated in tumor tissues when compared with the surrounding non-tumor counterparts (Figure 1A). To explore the clinical relevance of CPSF6 in GC patients, we next compared the expression of CPSF6 mRNA, with the RNA-seq data derived from GC (*n* = 352) and non-tumor tissues (*n* = 32) in TCGA. We discovered that the expression of CPSF6 was significantly upregulated in GC tissues (Figure 1B). According to 7th edition of the AJCC Cancer Staging Manual, cancer stages and nodal metastasis were recommended to the TNM gastric cancer staging system (Washington, 2010). To further examine the levels of CPSF6 in distinct GC stages and nodal metastasis, we evaluated the expression of CPSF6 in normal and GC tissues based on individual cancer stages or nodal metastasis. The results showed that the levels of CPSF6 were higher in GC. However, the levels of CPSF6 in different stages and nodal metastasis were similar (Figures 1C,D and Supplementary Tables 1, 2). We also analyzed the overall survival curves of CPSF6, and the result showed no significant difference between low and high level of CPSF6 in GC patients (data not shown). The malignancy of GC exhibits the following characteristics: metastasis and uncontrolled proliferation (Hu et al., 2020). The results implied that involvement of CPSF6 in GC formation may be due to the cell growth, rather than the metastasis in GC. We then examined the protein expression of CPSF6 in non-tumorous gastric cell line GES1 and human GC cell lines (AGS, BGC-823, MKN-28). We found that the protein levels of CPSF6 increased in the GC cell lines compared with non-tumorous gastric cell line (Figure 1E). Together, our data suggested that CPSF6 was upregulated in GC tissue and cells.

### CPSF6 Promotes Cell Growth and Inhibits Apoptosis in GC Cells *in vitro*

**FIGURE 2:**
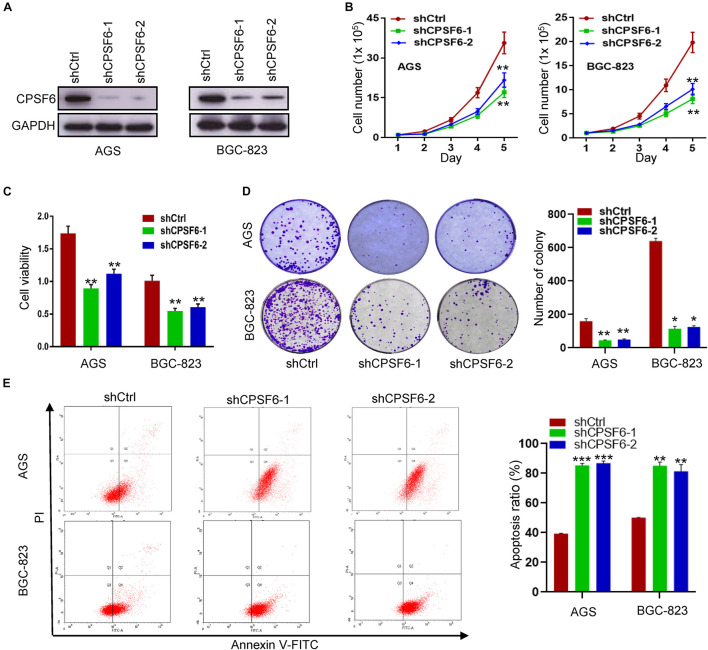
CPSF6 promotes cell growth and inhibits apoptosis in GC cells *in vitro*. **(A)** Western blotting analysis of CPSF6 expression in AGS and BGC-823 cells stably transfected with shRNA plasmids. **(B,C)** Detection of cell proliferation and viability after knockdown of CPSF6 in AGS and BGC-823 cells. **(D)** Detection of colony formation after knockdown of CPSF6 in AGS and BGC-823 cells. **(E)** Detection of apoptosis in AGS and BGC-823 cells. *0.01 < *p* < 0.05; **0.001 < *p* < 0.01; ***p < 0.001.

To investigate the function of CPSF6 in GC, we knocked down CPSF6 with short hairpin RNAs (shRNAs) in AGS and BGC-823 cells, which have higher levels of CPSF6 (Figure 1E). Expression of CPSF6 in these cell lines was obviously repressed (Figure 2A). Compared with the control, CPSF6 knockdown in AGS and BGC-823 significantly decreased cell proliferation (Figure 2B), viability (Figure 2C), and generated less and smaller colonies in colony formation assay (Figure 2D). It is reported that the cell cycle and apoptosis affect cell proliferation (Alenzi, 2004). We next investigated if CPSF6 regulated the cell growth through cell cycle and apoptosis in GC cells. The results showed that AGS-shCPSF6 cells increased fraction of S phase cells (*p* < 0.001), but BGC-823-shCPSF6 cells showed no significant changes (*p* > 0.05) (Supplementary Figure 1). Interestingly, both CPSF6 knockdown cell lines exhibited noteworthy increases in cell apoptosis (Figure 2E).

### CPSF6 Promotes Proliferation and Inhibits Apoptosis of GC Cells *in vivo*

**FIGURE 3:**
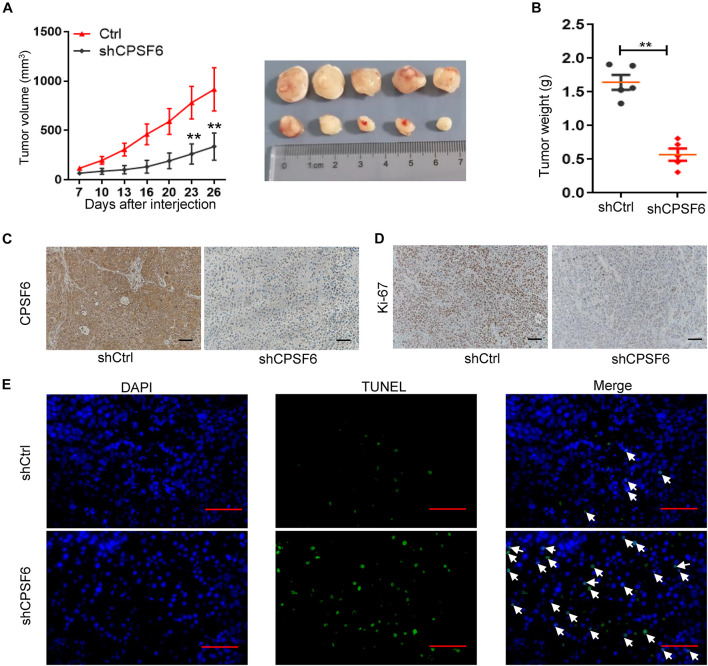
CPSF6 promotes cell growth and inhibits apoptosis in GC cells *in vivo*. **(A,B)** Xenograft nude mouse model of tumorigenesis was used to confirm cell growth and weight after knockdown of CPSF6. **(C,D)** CPSF6 and Ki-67 staining of the respective GC cell–derived tumor sections (scale bar: 100 μm). **(E)** TUNEL assay of the respective GC cell–derived tumor sections (scale bar: 100 μm). The white arrows represent apoptotic cells. (**0.001 < *p* < 0.01).

Considering that AGS cells have the highest levels of CPSF6 in the three GC cell lines, the AGS stably transfected cells (AGS-shCtrl, AGS-shCPSF6) were implanted subcutaneously into the flanks of nude mice to further assess the role of CPSF6 *in vivo*. CPSF6 silencing mouse model result also showed similar biological effects in GC cells. We observed a significant decrease in both tumor growth and weight (Figures 3A,B) in AGS-shCPSF6 mouse model compared with shCtrl tumors. Besides that, Ki-67 staining result displayed that tumors derived from AGS-shCPSF6 cells exhibited a significantly lower percentage of proliferative cells, as opposed to tumors derived from AGS-shCtrl cells (Figures 3C,D). In contrast, TUNEL assay result showed that CPSF6 knockdown increased the apoptosis percentage (Figure 3E). Collectively, our results demonstrated that knockdown of CPSF6 inhibited cell growth and increased apoptosis in GC cells.

### Knockdown of CPSF6 Modulates the Widespread 3′UTR Shortening in GC Cells

**FIGURE 4:**
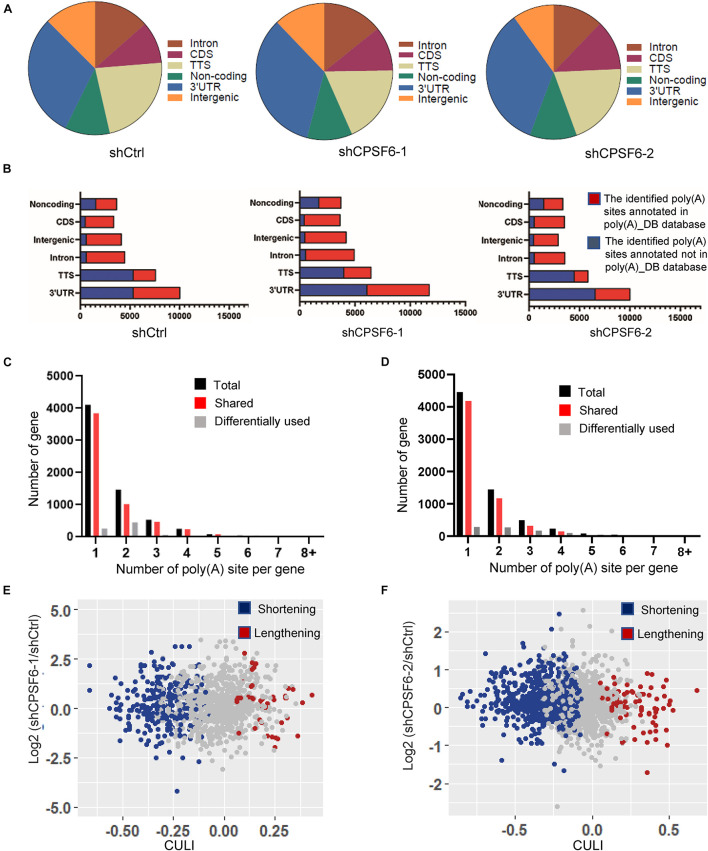
Characteristics of 3T-seq data. **(A)** Genomic locations of 3T-seq reads mapped to the reference genome after filtering internal priming events. **(B)** Genomic distribution of the poly(A) sites. **(C,D)** The statistics of genes with various number of detected poly(A) sites in different libraries. **(E,F)** Scatterplot of CULI in CPSF6 knockdown and control cells [false discovery rate (FDR) < 0.05] in different libraries.

Given that CPSF6 involves in APA formation and its role in promoting cell growth and restraining apoptosis in GC cells as described earlier, we hypothesized that CPSF6 acts as a tumor promoter in GC, at least in part, by influencing APA and 3′UTR. To test this hypothesis, we performed transcriptome-wide APA profiling analysis in CPSF6 knockdown AGS cells and the control cells, using 3T-seq we reported previously (Lai et al., 2015). In total, we gained about 35.7 million reads in AGS-shCtrl cells, as well as 28.9 and 30.9 million reads in AGS-shCPSF6-1 and shCPSF6-2 cells, respectively. About 10.4, 6.6, and 9.1 million reads were uniquely mapped to the reference genome (Supplementary Table 3). The internal priming was determined through examining the presence of more than 12 “A”s or continuous 8 “A”s at the region of 1–20 nt downstream the mapping position. After filtering internal priming events, we found majority of qualified reads were mapped to the annotated transcription terminal sites (TTSs) or 3′UTRs (Figure 4A), yielding over 10,000 poly(A) sites (Supplementary Table 3).

Applying the snowball clustering method (Fu et al., 2011), we found that most of the identified poly(A) sites were located at the 3′ terminus of genes. Particularly, about 60–76% of these poly(A) sites were mapped to TTSs and 52–70% to the 3′UTR regions in each 3T-seq library (Figure 4B), according to PolyA_DB database (Wang et al., 2018). More than one third of these poly(A) sites have been annotated in the poly(A) database, and thus the remaining sites may be potential new sites (Figure 4B). Also, about 30% of genes harbor three or more poly(A) sites (Figures 4C,D). We measured the 3′UTR alteration for CPSF6 responsive genes by introducing cancer 3′UTR length index (CULI) (Fu et al., 2011). A positive CULI suggests that a gene harbors lengthened 3′UTR in CPSF6 knockdown cells compared with the control cells, and a negative CULI indicates the shortened one. Compared with AGS-shCtrl cells, we identified 494 and 633 genes with altered 3′UTR in two independent CPSF6 shRNA knockdown AGS cells, respectively, and 83% and 89% of which displayed a shift from distal to proximal poly(A) site usage and thus possessed shortened 3′UTRs (Figures 4E,F and Supplementary Tables 4, 5). This result revealed that knockdown of CPSF6 modulated the widespread 3′UTR shortening in GC cells, which was consistent with previous studies in different cell types (Gruber et al., 2012; Martin et al., 2012).

### CPSF6 Downregulates the Expression of VHL Through APA

**FIGURE 5:**
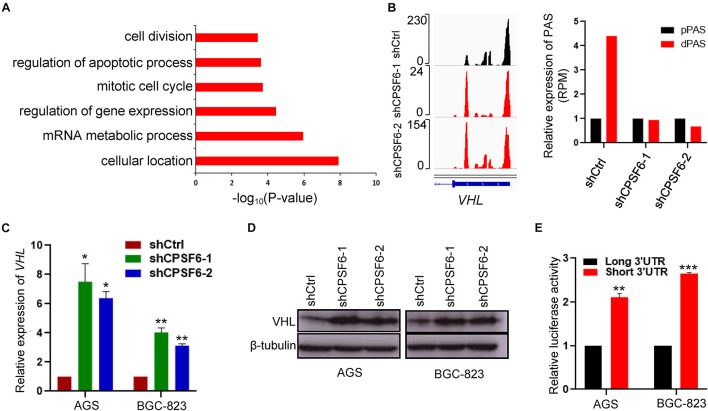
CPSF6 downregulates the expression of VHL. **(A)** GO analysis of genes with shortened 3′UTR after knockdown of CPSF6 in AGS cells. **(B)** CPSF6 induced APA shift of *VHL*. Left, Integrative Genomics Viewer (IGV) genome browser exhibited the poly(A) site usage of *VHL* 3′UTR. Right, histogram showed the relative expression of the isoform with distal polyadenylation site (dPAS) relative to the one with proximal PAS (pPAS). **(C)** The mRNA levels of *VHL* in CPSF6 knockdown AGS and BGC-823 cells. **(D)** The protein levels of VHL in CPSF6 knockdown AGS and BGC-823 cells. **(E)** The luciferase reporter assay for the expression activity of the isoform of *VHL* 3′UTR in AGS and BGC-823 cells. *0.01 < *p* < 0.05; **0.001 < *p* < 0.01; ***p < 0.001).

To ask how CPSF6-modulated APA contributes GC tumorigenesis, we assessed the functional consequence of CPSF6 responsive genes. Thus, to further understand the biological function of APA alternation in GC cells, we collected the 243 genes with shortened 3′UTR, which was simultaneous happened in the two independent AGS CPSF6 knockdown cells and performed the gene ontology (GO) enrichment analysis, using PANTHER classification system (*p-*value < 0.001, FDR < 0.005)^4^ (Supplementary Table 6). Notably, GO terms indicated that 3′UTR shortened genes are involved in regulation of apoptosis process and in other biological processes such as cellular and mRNA metabolic processes (Figure 5A).

Next, we manually inspected the apoptosis-promoting or proliferation-inhibiting genes under the criterion that the proximal poly(A) site usage increased and the distal poly(A) site usage decreased in both AGS knockdown cells, which were visualized in Integrative Genomics Viewer (IGV) genome browser, yielding three candidates (*IER3IP1*, *IGF1R*, and *VHL*) with top 3′UTR usage fold changes (Figure 5B and Supplementary Figure 2A). Then, we detected the mRNA expression of the candidate genes by RT-qPCR, and identified *VHL* as CPSF6 responsive gene, which had highest increased mRNA levels in CPSF6 knockdown AGS and BGC-823 cells (Figure 5C and Supplementary Figure 2B). Moreover, western blotting results indicated that CPSF6 negatively regulated VHL, that is, VHL expression increased in case of CPSF6 knockdown (Figure 5D).

A recent study has shown that the gene isoform with short 3′UTR had higher protein expression than the longer one, via escaping the repression of miRNA (Sandberg et al., 2008). As predicted, most of the predicted miRNA binding sites lay outside the proximal poly(A) sites on *VHL* 3′UTR (Supplementary Figure 2C). To investigate if the distinct 3′UTR isoforms affect the expression of proteins, we examined expression the short or long *VHL* 3′UTR fragments through the dual luciferase reporter system. As expected, the vector with short 3′UTR showed significantly higher luciferase activities than that with the long 3′UTR, which means short 3′UTR had higher expression ability (Figure 5E). Taken together, these data suggested that the expression of VHL was downregulated by CPSF6 through APA in GC.

### *VHL* Short−3′UTR Isoform Enhances Apoptosis and Inhibits Cell Growth in GC Cells

**FIGURE 6:**
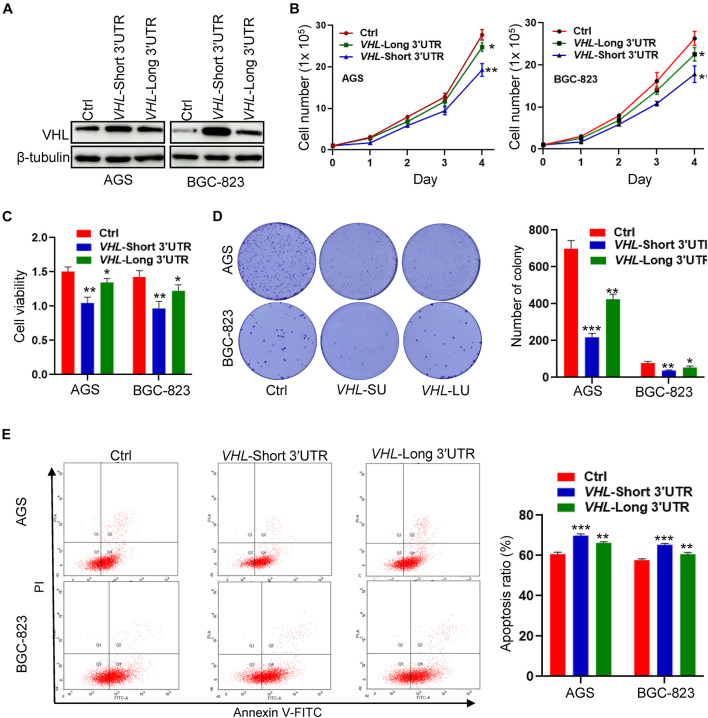
The *VHL* isoform with short 3′UTR inhibits cell growth and promotes apoptosis in GC cells. **(A)** Western blotting analysis of VHL expression in AGS and BGC-823 cells stably transfected with distinct *VHL* isoform with short or long 3′UTR. **(B,C)** Detection of cell proliferation and viability after stably enforced expression of the short- or long-3′UTR isoform of *VHL* in AGS and BGC-823 cells. **(D)** Detection of colony formation after stably enforced expression of the *VHL* isoform with short or long 3′UTR in AGS and BGC-823 cells. **(E)** Apoptosis ratio was analyzed in AGS and BGC-823 cells. *0.01 < *p* < 0.05; **0.001 < *p* < 0.01; ***p < 0.001.

To study the biological function of distinct *VHL* isoforms in GC cells, we stably transfected short- or long−3′UTR isoform of *VHL* into two GC cell lines: AGS and BGC-823. The protein expression of VHL is shown in Figure 6A. We found that the short−3′UTR *VHL* isoform dramatically upregulated the protein expression of VHL, as compared with transfection with the long−3′UTR *VHL* isoform. We further observed that of the short−3′UTR isoform of *VHL* in GC cells significantly decreased cell growth, cell viability (Figures 6B,C), colony formation (Figure 6D), and enhanced cell apoptosis (Figure 6E) when compared with that of control cells with the expression of full-length *VHL* and control vector. These data, collectively, suggested high levels of protein produced by *VHL* with shorter 3′UTR may, at least partially, contribute to enhance apoptosis and inhibit cell growth in GC cells.

## Discussion

GC is the third leading cause of cancer mortality across the world (Zhang and Zhang, 2017; Smyth et al., 2020). However, the mechanisms of GC progression are poorly understood. Increasing evidence has proved that APA is involved in many biological functions, including tumorigenesis (Singh et al., 2009; Xia et al., 2014). The APA-mediated regulatory mechanism is determined by numerous APA factors (Tian and Manley, 2017), and some of them are reported to participate in the tumor process. For example, NUDT21 showed a dual role in different cancer types (Masamha et al., 2014; Zhang and Zhang, 2018). This reflects that the regulatory mechanism of APA factors is complex and cancer specific, and it is necessary to characterize the role of APA in different types of cancers. Although CPSF6 played roles in aggressive breast cancer behavior and exhibited a novel CPSF6-RARG fusion variant in acute myeloid leukemia patients (Binothman et al., 2017; Zhang et al., 2020), the role of CPSF6 in GC is still unknown.

In this study, we examined expression of CPSF6 in GC and normal tissues. We found that CPSF6 was upregulated in GC, and what is more, the levels of CPSF6 in different stages and nodal metastasis were similar. We also analyzed the survival curves of CPSF6, and the result showed no significant difference between low and high level of CPSF6 in GC patients (data not shown). This may imply CPSF6 is not involved in metastasis and may contribute to other biological processes (such as proliferation) during GC initiation and progress. Here, we focus on investigating the relationship between CPSF6 and GC growth. Through *in vitro* and *in vivo* assays, we demonstrated CPSF6 enhances the proliferation and inhibits apoptosis in GC.

Given that CPSF6 involves in APA formation and its relevance in GC cells as described previously, we hypothesized that CPSF6 acts as a tumor promoter in GC, at least in part, by influencing APA and 3′UTR. We next performed the APA profiling analysis and identified the majority of APA genes with shortened 3′UTR under the condition of CPSF6 knockdown. The data suggested that knockdown of CPSF6 modulates the widespread 3′UTR shortening in GC cells. In another of our study, we analyzed the usage of 3′UTR in GC relative to normal cells (Lai et al., 2015), and the results showed a global 3′UTR shortening and tendency to use shorter isoforms in GC relative to normal cells. It is very interesting that cancer is associated with global 3′UTR shortening relative to normal cells, and our results seem that CPSF6 promotes cancer via 3′UTR lengthening. Consider that APA factors involve in cancer and regulate the APA progress. We have analyzed the expression of 20 core APA factors in GC and normal tissues using a website tool (see text footnote 3). Beyond our expectation, we found that 19 of 20 core APA factors were upregulated in GC tissues, and only RBP1 was not regulated significantly (*p* = 0.109) (data not shown). Next, we have searched the function of the 19 APA factors on regulation of 3′UTR. We found that eight factors (CPSF1, CPSF3, CSTF3, CSTF1, PCF11, SYMPK, RBBP6, FIP1L1) prefer to produce the short 3′UTR, and the rest of the factors (WDR33, CPSF4, CPSF2, CSTF2, NUDT21, CPSF6, CPSF7, CLP1, PAPOLA, PABPN1, PABPC1, RBP1) prefer to produce the long 3′UTR (Gruber et al., 2012; Martin et al., 2012; Li et al., 2015). Different factors make different contributions to APA. So, the tendency to use which isoforms in GC is determined by multiple factors. We assumed that although CPSF6 could promote cancer via 3′UTR lengthening, the other APA factors still regulated 3′UTR shortening in GC cells and led GC cells to still prefer to use short 3′UTR.

To understand how CPSF6 exerts its proliferation-promoting and apoptosis-inhibiting function by APA in GC cells, we further characterized CPSF6 regulated genes with shortened 3′UTRs. VHL was demonstrated to contribute to the function of CPSF6-mediated tumor growth in GC cells. We also analyzed the expression of *VHL* in GC relative to normal tissues. The results showed that the levels of *VHL* were higher in GC tissues and the expression of *VHL* in different stages and nodal metastasis was similar (data not shown). As CPSF6 was upregulated in GC cells and downregulated in the *VHL*, how was *VHL* increased in GC tissues? As we all know, many genes are expressed in cancer abnormally, and each of these genes makes partial contribution to cancer. To figure out this question, we analyzed the expression of all genes in GC relative to normal tissues (data not shown). We found that about 3,740 genes were upregulated in GC tissues, including 19 APA factors as described earlier. The 19 APA factors may play an important role in regulating the 3′UTR of *VHL* in different ways and lead to higher expression of *VHL* in GC. Other genes were also increased in GC tissues, for example, *ZNF350* (*ZBRK1*) was upregulated in GC tissues, and it was reported that *ZNF350* could activate *VHL* gene transcription through formation of a complex with *VHL* and p300 in renal cancer (Chen et al., 2015). We also found that about 900 genes were downregulated in GC tissues, including *Daam2*. According to the report, *Daam2* suppressed *VHL* expression and promoted tumorigenesis (Zhu et al., 2017). We supposed that although CPSF6 negatively regulated the *VHL* expression partly, other proteins might increase the level of *VHL* in GC tissues, and which was stronger than the effect of CPSF6 on expression of *VHL*, leading to a higher level of *VHL* in GC tissues.

**FIGURE 7:**
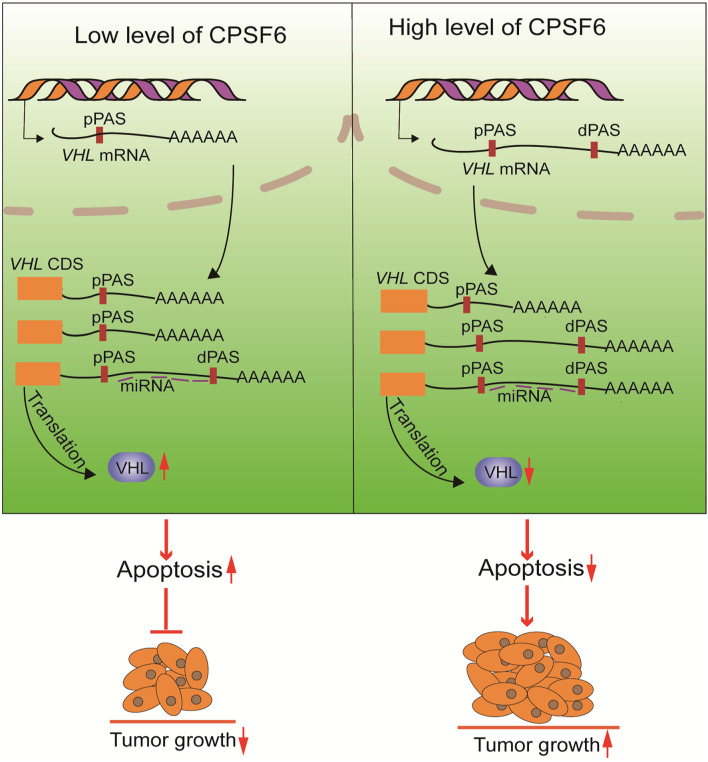
The proposed mechanism of CPSF6 in tumorigenesis of GC progression.

In this study, we validated that knockdown of CPSF6 leads to a shift from distal to proximal poly(A) site usage, the shortening of 3′UTR in *VHL*, which consequently causes the increased expression of VHL, and inhibition of proliferation in GC cells (Figure 7). *VHL* functions as a tumor suppressor gene in some tumors, such as renal cell carcinoma and pheochromocytoma (Richards, 2001), but few studies have reported its role in GC. Interestingly, it was reported that the genetic and epigenetic alterations of the *VHL* were not detected in GC (Cao et al., 2008). Thus, our study may offer a new perspective to understand the regulation of *VHL*. However, how CPSF6 regulates the 3′UTR length and whether other APA factors are involved this progress and the regulation of APA factors in GC and normal tissues still need to be explored. In any case, our data may provide new insights into the understanding of CPSF6’s role in GC cells and may imply potential therapeutic targets.

## Data Availability Statement

The datasets presented in this study can be found in online repositories. The names of the repository/repositories and accession number(s) can be found in the article/Supplementary Material.

## Ethics Statement

The studies involving human participants were reviewed and approved by Biomedical Ethics Committee of Anhui Medical University. The patients/participants provided their written informed consent to participate in this study. The animal study was reviewed and approved by Institutional Animal Care and Use Committee, Shanghai Jiao Tong University.

## Author Contributions

XS, KD, and ST performed most of the experiments and interpretation of the data. QZ, PL, and YK contributed to the analysis of the data and advised on the experimental design. ST and XS wrote the article. ST and JS critically revised the manuscript and contributed to the conception and design. All authors read and approved the final article.

## Conflict of Interest

The authors declare that the research was conducted in the absence of any commercial or financial relationships that could be construed as a potential conflict of interest.

## Publisher’s Note

All claims expressed in this article are solely those of the authors and do not necessarily represent those of their affiliated organizations, or those of the publisher, the editors and the reviewers. Any product that may be evaluated in this article, or claim that may be made by its manufacturer, is not guaranteed or endorsed by the publisher.

## Footnotes

1
http://www.ebi.ac.uk/arrayexpress

2
https://www.cancer.gov/tcga

3
http://ualcan.path.uab.edu/analysis.html

4
http://geneontology.org

## Supplementary Material

Supplementary Figure 1 The cell cycle distribution after knockdown of CPSF6 in AGS and BGC-823 cells.

Supplementary Figure 2 The candidate genes of CPSF6. **(A)** CPSF6 induced APA shift of candidate genes. UP, Integrative Genomics Viewer (IGV) genome browser exhibited the poly(A) site usage of candidate genes’ 3′UTR. Down, histogram showed the relative expression of the isoform with distal polyadenylation site (dPAS) relative to the one with proximal PAS (pPAS). **(B)** The mRNA levels of candidate genes in CPSF6 knockdown AGS cells (up) and BGC-823 cells (down). **(C)** Schematic illustration of the *VHL* isoform with long or short 3′UTR. Positions of the binding sites of miRNA were predicted by website tool (http://www.targetscan.org/) and indicated by purple horizontal lines. CR, CDS region. ^∗^0.01 < *p* < 0.05; ^∗∗^0.001 < *p* < 0.01; ^∗∗∗^*p* < 0.001.

Supplementary Table 1 Statistical significance of CPSF6 expression in GC of individual cancer stages.

Supplementary Table 2 Statistical significance of CPSF6 expression in GC based on nodal metastasis status.

Supplementary Table 3 The sequencing statistics of AGS 3T-seq libraries.

Supplementary Table 4 The list of genes with altered 3′UTR in AGS shCPSF6-1 cells.

Supplementary Table 5 The list of genes with altered 3′UTR in AGS shCPSF6-2 cells.

Supplementary Table 6 The list of genes with shortened 3′UTR shared by AGS shCPSF6-1 and shCPSF6-2 cells.
