# Supplementary figures and images for: Suppression of CPSF6 Enhances Apoptosis Through Alternative Polyadenylation-Mediated Shortening of the VHL 3′UTR in Gastric Cancer Cells
Source: Front Genet. 2021 Sep 14;12:707644. doi: 10.3389/fgene.2021.707644 (PMC8477001; doi:10.3389/fgene.2021.707644)

AGS

shCtrl

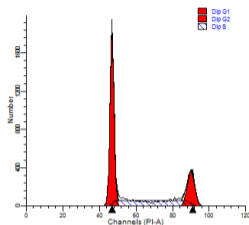

shCPSF6-1

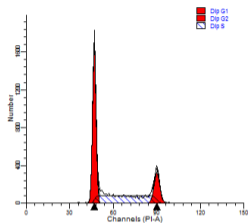

shCPSF6-2

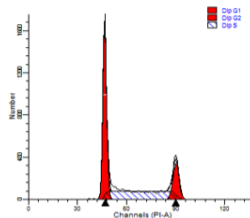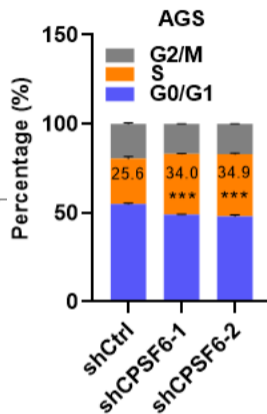

BGC-823

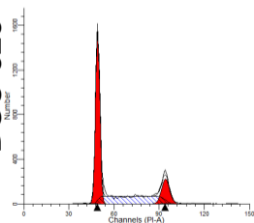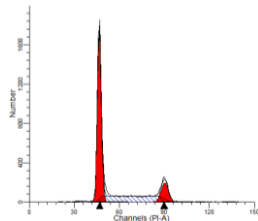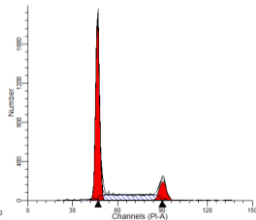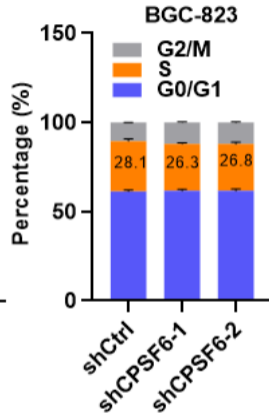

Supplement: Supplementary Figure 1 — The cell cycle distribution after knockdown of CPSF6 in AGS and BGC-823 cells. [file Data_Sheet_1.PDF]

a

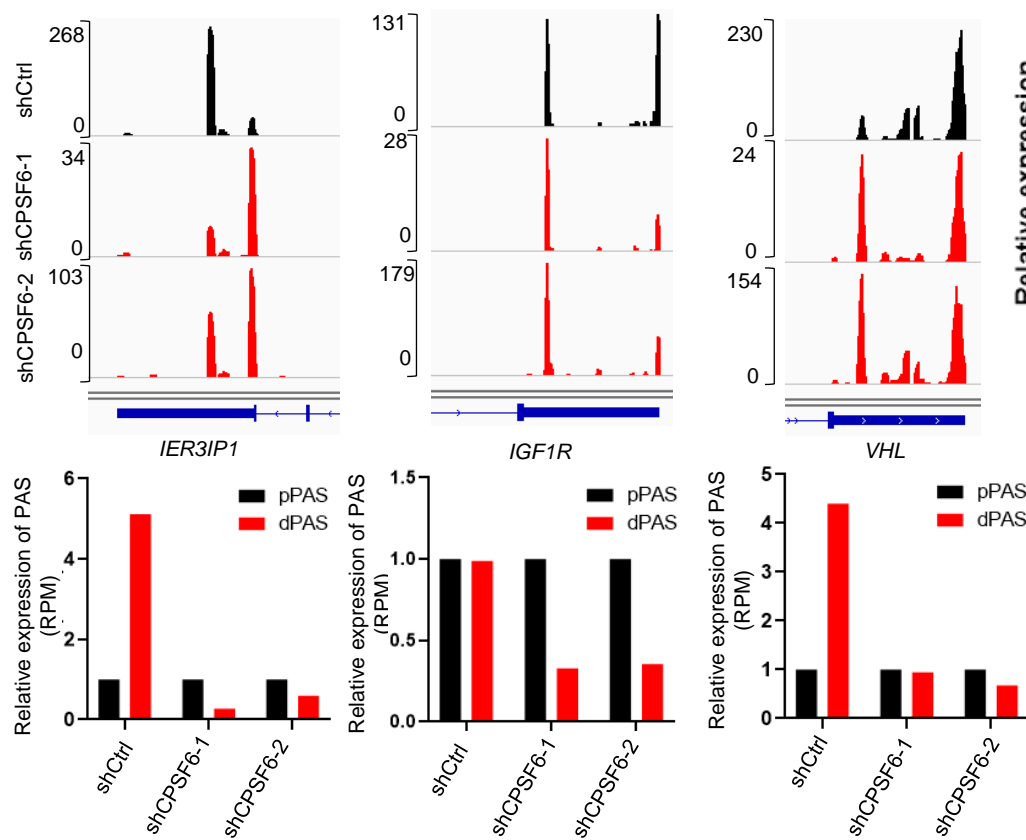

b

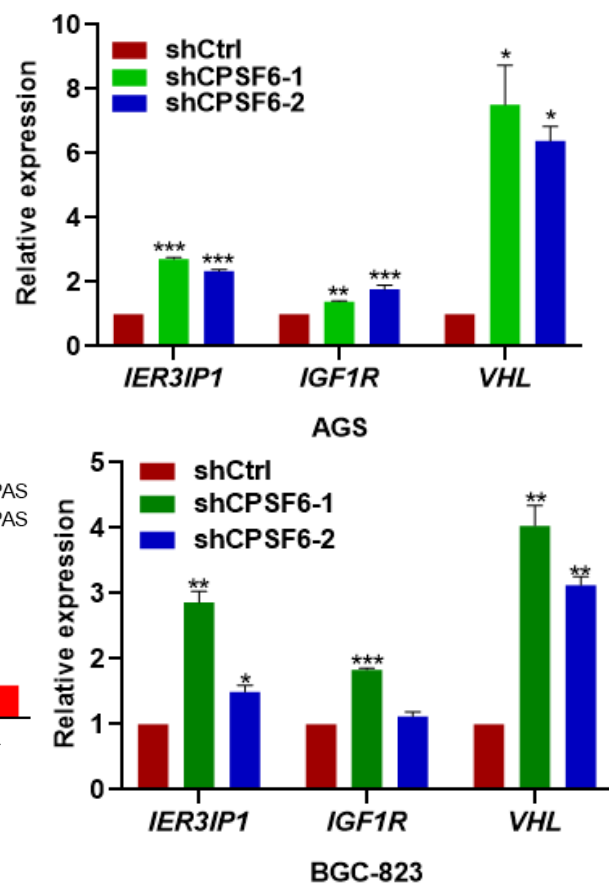

c

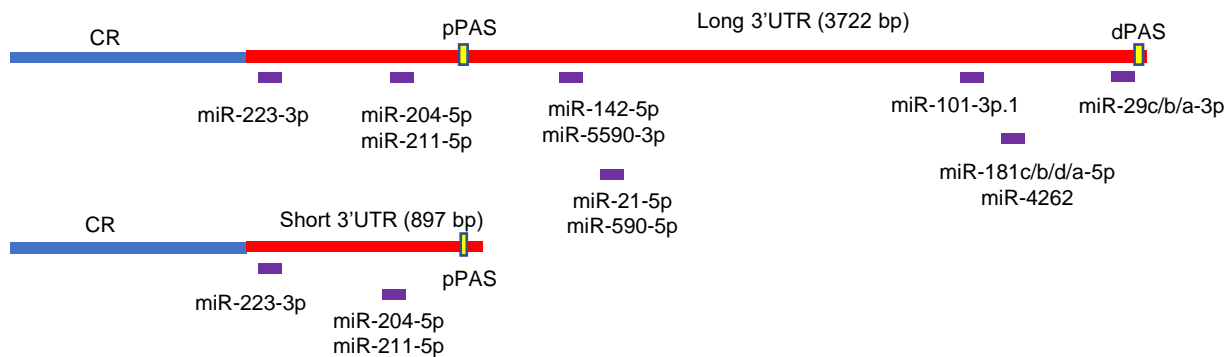

Supplement: Supplementary Figure 2 — The candidate genes of CPSF6. (A) CPSF6 induced APA shift of candidate genes. UP, Integrative Genomics Viewer (IGV) genome browser exhibited the poly(A) site usage of candidate genes’ 3′UTR. Down, histogram showed the relative expression of the isoform with distal polyadenylation site (dPAS) relative to the one with proximal PAS (pPAS). (B) The mRNA levels of candidate genes in CPSF6 knockdown AGS cells (up) and BGC-823 cells (down). (C) Schematic illustration of the VHL isoform with long or short 3′UTR. Positions of the binding sites of miRNA were predicted by website tool (http://www.targetscan.org/) and indicated by purple horizontal lines. CR, CDS region. ∗0.01 < p < 0.05; ∗∗0.001 < p < 0.01; ∗∗∗p < 0.001. [file Data_Sheet_2.PDF]
