# Supplementary material for: Suppression of CPSF6 Enhances Apoptosis Through Alternative Polyadenylation-Mediated Shortening of the VHL 3′UTR in Gastric Cancer Cells
Source: Front Genet. 2021 Sep 14;12:707644. doi: 10.3389/fgene.2021.707644 (PMC8477001; doi:10.3389/fgene.2021.707644)
Supplement: Supplementary Table 1 — Statistical significance of CPSF6 expression in GC of individual cancer stages. [file Table_1.DOCX]

**Supplementary Table 1** Statistical significance of CPSF6 expression in GC of individual cancer stages

| Comparison | Statistical significance (*P*) |
| --- | --- |
| Normal-vs-Stage1 | 1.68E-05 |
| Normal-vs-Stage2 | 1.11E-16 |
| Normal-vs-Stage3 | 1.62E-12 |
| Normal-vs-Stage4 | 2.18E-03 |
| Stage1-vs-Stage2 | 9.59E-01 |
| Stage1-vs-Stage3 | 4.08E-01 |
| Stage1-vs-Stage4 | 1.97E-01 |
| Stage2-vs-Stage3 | 1.09E-01 |
| Stage2-vs-Stage4 | 1.89E-01 |
| Stage3-vs-Stage4 | 3.20E-01 |

Significance of difference estimated by Student’s t-test considering unequal variance.
