# Supplementary material for: Suppression of CPSF6 Enhances Apoptosis Through Alternative Polyadenylation-Mediated Shortening of the VHL 3′UTR in Gastric Cancer Cells
Source: Front Genet. 2021 Sep 14;12:707644. doi: 10.3389/fgene.2021.707644 (PMC8477001; doi:10.3389/fgene.2021.707644)
Supplement: Supplementary Table 2 — Statistical significance of CPSF6 expression in GC based on nodal metastasis status. [file Table_2.DOCX]

**Supplementary Table 2** Statistical significance of CPSF6 expression in GC based on nodal metastasis status

| Comparison | Statistical significance |
| --- | --- |
| Normal-vs-N0 | 1.62E-12 |
| Normal-vs-N1 | <1E-12 |
| Normal-vs-N2 | 1.62E-12 |
| Normal-vs-N3 | 7.83E-07 |
| N0-vs-N1 | 1.35E-01 |
| N0-vs-N2 | 1.44E-01 |
| N0-vs-N3 | 2.14E-01 |
| N1-vs-N2 | 7.89E-01 |
| N1-vs-N3 | 6.48E-01 |
| N2-vs-N3 | 5.36E-01 |

Significance of difference estimated by Student’s t-test considering unequal variance. Pathologic N descriptions: N0, No regional lymph node metastasis; N1, metastases in 1 to 3 axillary lymph nodes; N2, metastases in 4 to 9 axillary lymph nodes; N3, metastases in 10 or more axillary lymph nodes.
