# Supplementary material for: Suppression of CPSF6 Enhances Apoptosis Through Alternative Polyadenylation-Mediated Shortening of the VHL 3′UTR in Gastric Cancer Cells
Source: Front Genet. 2021 Sep 14;12:707644. doi: 10.3389/fgene.2021.707644 (PMC8477001; doi:10.3389/fgene.2021.707644)
Supplement: Supplementary Table 3 — The sequencing statistics of AGS 3T-seq libraries. [file Table_3.DOCX]

**Supplementary Table 3** The sequencing statistics of AGS 3T-seq libraries

| Library  Items | shCtrl | shCPSF6-1 | shCPSF6-2 |
| --- | --- | --- | --- |
| Raw reads | 35,704,544 | 28,907,034 | 30,864,866 |
| Total reads after trimming | 30,474,029 | 26,575,751 | 27,512,312 |
| Reads mapped to genome | 10,403,869 | 6,592,184 | 9,105,092 |
| Reads passed internal priming filter | 7,019,758 | 3,946,224 | 6,143,162 |
| Identified poly(A) sites (merged) | 10,641 | | |
| Genes hit by poly(A) sites (merged) | 6,530 | | |
